# SUPPORTING AGING IN PLACE THROUGH IWISH: LESSONS FROM THE SUPPORTIVE SERVICES DEMONSTRATION

**DOI:** 10.1093/geroni/igae098.2959

**Published:** 2024-12-31

**Authors:** Melissa Vandawalker, Elizabeth Giardino, Meghan Hendricksen, Ian Breunig

**Affiliations:** Abt Global, Cambridge, Massachusetts, United States; Abt Global, Cambridge, Massachusetts, United States; Abt Global, Cambridge, Massachusetts, United States; Abt Global, Nashville, Tennessee, United States

## Abstract

Most Americans prefer to live independently in their homes as long as possible. The U.S. Congress sponsored the Supportive Services Demonstration to learn whether the Integrated Wellness in Supportive Housing (IWISH) model can help adults aged 62 and older remain in HUD-assisted housing developments longer and delay transitions to long-term care. After three years of the demonstration, we found little evidence that IWISH affected residents’ tenure or healthcare utilization as intended, but there are reasons for optimism about IWISH’s long-term impact and lessons learned from 40 properties that implemented IWISH between 2017–2020. Based on enrollment and services data for 2,927 IWISH participants, 21 focus groups with 180 residents, and interviews with property management and onsite wellness staff, we conducted an implementation analysis of IWISH properties’ fidelity to the model and identified IWISH’s strengths and weaknesses. We observed substantial variation in resident participation and how properties implemented the model. We identified challenges with hiring and retaining onsite wellness staff for a diverse multilingual resident population, developing partnerships with local healthcare organizations, and addressing residents’ privacy concerns over their health information. Residents and staff reported numerous benefits from IWISH suggesting a promising strategy for helping residents age in place, including examples of preventative actions by IWISH staff that helped residents avoid unnecessary emergency care, transitional care after returning from the hospital, and feeling safe and secure with a dedicated, onsite medical professional.

